# Endometrial compaction shows no association with improved pregnancy outcomes in hormonal replacement frozen-thawed embryo transfer: an analysis of over 16 000 cases

**DOI:** 10.1093/hropen/hoaf039

**Published:** 2025-06-20

**Authors:** Peipei Pan, Chang Liu, Shiyi Lin, Haiqing Wang, Xia Chen, Haiyan Yang, Xuefeng Huang, Huan Zhang, Yili Teng

**Affiliations:** Reproductive Medicine Center, The First Affiliated Hospital of Wenzhou Medical University, Wenzhou, Zhejiang, China; Reproductive Medicine Center, The First Affiliated Hospital of Wenzhou Medical University, Wenzhou, Zhejiang, China; Traditional Chinese Medicine Department, The Second Affiliated Hospital and Yuying Children’s Hospital of Wenzhou Medical University, Wenzhou, Zhejiang, China; Reproductive Medicine Center, The First Affiliated Hospital of Wenzhou Medical University, Wenzhou, Zhejiang, China; Reproductive Medicine Center, The First Affiliated Hospital of Wenzhou Medical University, Wenzhou, Zhejiang, China; Reproductive Medicine Center, The First Affiliated Hospital of Wenzhou Medical University, Wenzhou, Zhejiang, China; Reproductive Medicine Center, The First Affiliated Hospital of Wenzhou Medical University, Wenzhou, Zhejiang, China; Reproductive Medicine Center, The First Affiliated Hospital of Wenzhou Medical University, Wenzhou, Zhejiang, China; Reproductive Medicine Center, The First Affiliated Hospital of Wenzhou Medical University, Wenzhou, Zhejiang, China; The First Clinical Medical College, Shandong University of Traditional Chinese Medicine, Jinan, Shandong, China

**Keywords:** endometrial thickness, compacted endometrium, frozen-thawed embryo transfer, live birth rate, clinical pregnancy rate, transvaginal ultrasound

## Abstract

**STUDY QUESTION:**

Is there an association between changes in endometrial thickness (EMT) following progesterone administration and pregnancy outcomes in frozen-thawed embryo transfers (FETs) at Day 3 (D3) and blastocyst stages?

**SUMMARY ANSWER:**

Endometrial compaction is not associated with better pregnancy outcomes.

**WHAT IS KNOWN ALREADY:**

Previous studies have shown conflicting results on the impact of EMT changes on FET outcomes.

**STUDY DESIGN, SIZE, DURATION:**

This study was a single-center retrospective cohort analysis of FETs from 1 January 2018 to 31 December 2022. A total of 9390 D3 FETs and 7063 blastocyst FETs were included during this period.

**PARTICIPANTS/MATERIALS, SETTING, METHODS:**

D3 FETs and blastocyst FETs were divided into three groups: compaction group, non-change group, and expansion group. The impact of EMT changes after progesterone administration on HCG-positive, pregnancy, ongoing pregnancy, live birth, and pregnancy loss rates were analyzed for D3 and blastocyst FETs. EMT on the progesterone administration day (defined as EMT1) and on embryo transfer (ET)day (defined as EMT2) was measured exclusively by transvaginal ultrasound. Inverse probability weighting (IPW) and stratified logistic regressions were conducted to reduce the effects of confounding factors.

**MAIN RESULTS AND THE ROLE OF CHANCE:**

After IPW adjustment, in D3 FETs, women with compacted endometrium had the lowest HCG-positive rates (*P *= 0.012), clinical pregnancy rates (*P *< 0.001), ongoing pregnancy rates (*P *< 0.001), and live birth rates (LBRs) (*P *< 0.001) among the three groups. Among HCG-positive cases, the compaction group had the highest ectopic pregnancy rates (3.5% vs 2.6% vs 1.6%; *P *= 0.015) and the lowest LBRs (65.8% vs 68.3% vs 71.4%; *P *= 0.018). Univariate logistic regressions found that LBRs were weakly associated with compacted endometrium [odds ratio (OR) 0.831, 95% CI: 0.696–0.993]. Logistic regressions with IPW revealed that the compaction group was not associated with higher odds of pregnancy outcomes, including HCG positive, clinical pregnancy, ongoing pregnancy, ongoing pregnancy, and live births compared to the non-change group. In contrast, the expansion group was associated with higher odds of live birth per ETs (OR 1.166, 95% CI: 1.070–1.271; *P *= 0.001), and live birth per HCG-positive cases (OR 1.160, 95% CI: 1.028–1.309; *P *= 0.016). In blastocyst FETs, women with compacted endometrium had the lowest HCG-positive rates (*P *= 0.001) and clinical pregnancy rates (*P *= 0.031). Logistic regressions with IPW adjustment found that compaction group was associated with lower odds of HCG positive (OR 0.813, 95% CI: 0.668–0.989, *P *= 0.039) compared to the non-change group. Additionally, LBRs increased with the rising change ratios of EMT after progesterone administration, but within a certain range (30% in D3 FETs and 50% in blastocyst FETs).

**LIMITATIONS, REASONS FOR CAUTION:**

This study only included retrospective analyses of untested embryos for FETs.

**WIDER IMPLICATIONS OF THE FINDINGS:**

Endometrial expansion in D3 FETs exhibited a positive association with improved LBRs, but not in blastocyst FETs. These findings suggest that embryo developmental stage-specific endometrial preparation protocols may enhance assisted reproductive outcomes.

**STUDY FUNDING/COMPETING INTEREST(S):**

This study was supported by National Natural Science Foundation of China (82201856), Basic Scientific Research project of Wenzhou Science and Technology Bureau (Y20220006), Wenzhou Key Laboratory of Reproduction and Genetics (2022HZSY0051), and Clinical Technology International Exchange Project of Zhejiang Provincial Medical Institutions. The authors have no conflicts of interest to declare.

**TRIAL REGISTRATION NUMBER:**

N/A.

WHAT DOES THIS MEAN FOR PATIENTS? This study looked into whether changes in the thickness of the womb lining after using progesterone—a hormone given during fertility treatment—might affect pregnancy success.

Currently, both patients and doctors are often worried if the womb lining gets too thick or too thin after progesterone administration. Sometimes, women are even told to delay the embryo transfer or take extra medicines to address these changes.

In this study, patients were divided into three groups based on the extent of change in the thickness of their womb lining after progesterone treatment. The researchers found that when the womb lining becomes thin following progesterone treatment, it could not improve the chances of pregnancy success. This insight could help guide treatment decisions and reduce uncertainties for patients undergoing IVF.

## Introduction

Embryo implantation is a complex process in which an activated blastocyst attaches to the receptive endometrium of the uterus (Muter *et al.*, 2023). Recently endometrial receptivity has been the focus of extensive research, and is considered to be underestimated. Some studies even reported that suboptimal endometrial receptivity and altered embryo–endometrial crosstalk account for approximately two-thirds of implantation failures (Craciunas *et al.*, 2019; Idelevich and Vilella, 2020). Endometrial receptivity is usually assessed by several sonographic parameters, including endometrial thickness (EMT), endometrial pattern, endometrial volume, endometrial blood flow, and endometrial wave-like activity (Craciunas *et al.*, 2019; Li *et al.*, 2023). Furthermore, EMT has been widely accepted as an indicator to predict endometrial receptivity (Craciunas *et al.*, 2019; Li *et al.*, 2023). Pregnancy outcomes are considered to reflect the status of endometrial receptivity due to the lack of gold-standard diagnostic tests for endometrial receptivity. Emerging evidence has revealed that an EMT greater than 7 mm is associated with higher clinical pregnancy rates and live birth rates (LBRs) (von Wolff *et al.*, 2018; Liao *et al.*, 2021), implying that thin endometrium may reflect suboptimal endometrial receptivity and predict poor pregnancy.

Recently, increasing studies have focused on the effects of EMT changes after progesterone administration on the pregnancy outcomes, since the first study found that endometrial compaction (decreased thickness) could predict optimal ongoing pregnancy rates (Haas *et al.*, 2019). Some of subsequential studies have concluded similarly that compressed endometrium was associated with better pregnancy outcomes, including implantation rates (Yaprak *et al.*, 2023), clinical pregnancy rates (Youngster *et al.*, 2022; Ju *et al.*, 2023; Yaprak *et al.*, 2023), ongoing pregnancy rates (Haas *et al.*, 2019; Zilberberg *et al.*, 2020; Kaye *et al.*, 2021; Youngster *et al.*, 2022), LBRs (Yaprak *et al.*, 2023). But some studies have showed no significant differences between EMT changes and pregnancy results (Huang *et al.*, 2020; Ye *et al.*, 2020; Riestenberg *et al.*, 2021; Olgan *et al.*, 2022; Shah *et al.*, 2022). Interestingly, expanded endometrium has also been demonstrated to be a reliable indicator of clinical pregnancy rates (Bu *et al.*, 2019; Jin *et al.*, 2021a). Given pregnancy loss results, a study reported by Li *et al.* (2022) found that compressed endometrium significantly reduced the incidence of ectopic pregnancy in women with EMT ≥8 mm. In addition, some studies have found changes in EMT are not associated with the incidence of miscarriage (Jin *et al.*, 2021a; Kaye *et al.*, 2021; Yaprak *et al.*, 2023). Thus, it is uncertain whether EMT changes after progesterone administration are associated with pregnancy outcomes and whether the changes in EMT could be regarded as an indicator of endometrial receptivity.

In order to determine whether endometrial compaction has an effect on the success of assisted reproductive technology, several systematic reviews and meta-analyses have been performed and found LBRs were comparable in cycles showing endometrial compaction or not (Chen *et al.*, 2023; Turkgeldi *et al.*, 2023; Feng *et al.*, 2024). These systematic reviews and meta-analyses have reported that one of possible explanations for the contradictory findings of previous studies lies in the method by which the EMT is measured (Chen *et al.*, 2023; Turkgeldi *et al.*, 2023; Feng *et al.*, 2024). Ultrasound examination (transvaginal ultrasound and transabdominal ultrasound) has become a routine method for endometrial assessment, due to its convenient and non-invasive characteristics. At present, transvaginal ultrasound has been found to be higher accuracy compared to transabdominal ultrasound on the endometrial evaluation. Some studies assessed EMT by transvaginal ultrasound at the end of follicular phase, and subsequently by transabdominal ultrasound on embryo transfer (ET) day (Haas *et al.*, 2019; Zilberberg *et al.*, 2020; Shah *et al.*, 2022; Ju *et al.*, 2023), three of which found a positive correlation between endometrial compaction and good pregnancy outcomes (Haas *et al.*, 2019; Zilberberg *et al.*, 2020; Ju *et al.*, 2023); meanwhile, others exclusively used transvaginal ultrasound measurement (Ye *et al.*, 2020; Jin *et al.*, 2021a,b; Kaye *et al.*, 2021; Riestenberg *et al.*, 2021), four of which failed to find the predictive effects of endometrial compaction (Ye *et al.*, 2020; Jin *et al.*, 2021a,b; Riestenberg *et al.*, 2021).

The primary purpose of the present study was to investigate the association between EMT changes and pregnancy outcomes (clinical pregnancy rates, ongoing pregnancy rates, LBRs, pregnancy loss rates) in hormone replacement frozen-thawed embryo transfers (FETs). The second aim of the study was to determine whether there was a differential impact on pregnancy outcomes based on the degrees of EMT changes. Inverse probability weighting (IPW) and logistic regressions were used to estimate the effects of EMT changes on the pregnancy outcomes. To our knowledge, this retrospective study was the largest sample research, including 16 453 FETs with transvaginal ultrasound for all endometrial measurements. Considering the differences between different endometrial preparation protocols, FETs with the standard hormone replacement treatment were enrolled to reduce the confounding factors that might affect the status of endometrium and ensure the consistency of the study.

## Materials and methods

### Sample selection and ethical approval

This was a single-center retrospective cohort study of 16 453 hormone replacement FET cycles, which were performed in the Reproductive Medicine Centre of the First Affiliated Hospital of Wenzhou Medical University from 1 January 2018 to 31 December 2022. The study was approved by the Ethics Committee of Reproductive Medicine of the First Affiliated Hospital of Wenzhou Medical University (NO: KY2023-R202). The exclusion criteria were as follows: (i) Age <20 or >45 years old; (ii) BMI >40 or <19 kg/m^2^; (iii) The first two cycles with EMT <7 mm on the day of progesterone administration; (iv) Pre-implantation genetic testing (PGT); (v) Anti-Müllerian hormone (AMH) <0.5 ng/ml; (vi) Genetic disorders; (vii) Untreated gynecological disease: hydrosalpinx, uterine polyps, submucosal fibroids, severe adenomyosis, uterine fibroids (>5 cm), and uterine malformations; (viii) Incomplete follow-up records; (ix) Females diagnosed with basic diseases, including hypertension, diabetes, immune diseases. In order to exclude the effects of ovarian reserve on the pregnancy results, females with ≥0.5 ng/ml of AMH were included. In this study, there were 353 cycles in which EMT was less than 7 mm at the end of the proliferative phase but was thicker than in any previous fresh or FETs. During the study period, only the first three FETs would be included in the analyses if the participants had more than three FETs, and the first two cycles of the same participants failed to have live births.

### Endometrial preparation protocols and ET

All participants received the same hormonal (estrogen and progesterone) protocol for endometrial preparations. Patients started hormonal therapy with exogenous estradiol on Days 3–6 of the menstrual cycles. In all cases, patients were given oral administration of 2 mg progynova (Bayer-Schering Pharma AG, Berlin, Germany) twice daily, which was increased after 4 days to 3 mg twice daily. A transvaginal ultrasound was performed using the SonoScape S15 (Kaili Bio-Medical Technology, Guangdong, China) to assess the EMT and pattern after 10 days of estradiol administration. If EMT was less than 8 mm but more than 7 mm, estrogen administration would be continuously increased to 4 mg twice daily with/without vaginal administration of Femoston (Abbott Biologicals B.V., Olst, the Netherlands) for another 5–7 days and during this period, the ultrasound monitoring was conducted two times. Once EMT was more than 8 mm, or was less than 8 mm but thicker than in any previous fresh or FET cycles, besides exogenous estrogen, 40 mg intramuscular progesterone would be injected once daily on the same day and 10 mg dydrogesterone (Abbott Biologicals B.V., Olst, the Netherlands) twice daily combined on the following morning, transforming the endometrium to secretary phase. On 3 and 5 days after progesterone administration, cleavage stage embryos and blastocysts were transferred into the uterus, respectively. The embryos chosen for transfer were based on the following sequence: good-grade blastocysts (AA, AB, or BA)>moderate-grade blastocysts (BB)>low-grade blastocysts>I-grade day 3 (D3) embryo or II-grade D3 embryo>III-grade D3 embryo. On the morning of ET day, the EMT was assessed by transvaginal ultrasound. All the ultrasound measurements were performed by different clinicians under standard operating procedures. In addition, 90 mg Crinone 8% gel (Merck Serono Ltd, Middlesex, UK) once per day replaced intramuscular progesterone administration. Then, 13 days after cleavage-stage ETs and 11 days after blastocyst transfers, the level of serum beta HCG was determined to assess the outcomes of FETs. Clinical pregnancy was confirmed by transvaginal ultrasound 4 weeks after ET.

### EMT assessment

EMT was defined as the maximal distance between the endometrial and myometrial junctions at about 1.5 cm from the uterine fundus through the median sagittal section of the uterus. The data of EMTs were recorded from patient files. The EMTs on the day of progesterone administration and on ET day were recorded as EMT1 and EMT2, respectively. Based on EMT change ratio ($\frac{\mathrm{EMT}2-\mathrm{EMT}1}{\mathrm{EMT}1}\times 100\%$), the patients were divided into three groups: the endometrial compaction group, endometrial non-change group, and endometrial expansion group. As reported in previous studies (Haas *et al.*, 2019; Riestenberg *et al.*, 2021), the criterions for endometrial compaction and endometrial expansion were both set as 5%. Cycles in which the EMT change ratio was less than ±5% were considered unchanged. According to univariate logistic regression analyses, FETs were further stratified by female age, EMT1, EMT2, and the degrees of EMT change ratios (<−15%, −15 ∼−5%,−5 ∼ 5%, 5 ∼ 15%, 15 ∼ 30%, 30 ∼ 50%, and >50%), and then analyzed. Our primary outcome was LBRs per ET cycles, and the secondary outcomes were LBRs per HCG-positive cases, HCG-positive rates per ET cycles, clinical pregnancy rates per ET cycles, ongoing pregnancy rates per ET cycles, and ectopic pregnancy rates per HCG-positive cases.

### Embryo grading and cryopreservation techniques

The quality of embryos at cleavage stage was graded according to the Istanbul consensus (Alpha Scientists in Reproductive Medicine and ESHRE Special Interest Group of Embryology, 2011). The quality of blastocysts was assessed according to Gardner scoring (Alpha Scientists in Reproductive Medicine and ESHRE Special Interest Group of Embryology, 2011). Blastocysts were classified with an A or B in both inner cell mass (ICM) and trophectoderm (TE) as good-grade blastocysts (AA, AB, or BA), those with a B in both ICM and TE as moderate-grade blastocysts (BB), and those with a C in either ICM or TE as low-grade blastocysts (AC, CA, BC, CB, CC) (Zou *et al.*, 2023). Embryos were cryopreserved using the vitrification technique. In the present study, none of them underwent PGT for aneuploidy (PGT-A).

### Definition of clinical outcomes

Pregnancy was defined as a serum beta HCG level >10 mIU/ml at 11–13 days following ET. Biochemical pregnancy was defined as a serum beta HCG level >10 mIU/ml without the presence of a gestational sac with a yolk sac in the uterus detected by transvaginal ultrasonography. Clinical pregnancy was defined as the presence of a gestational sac with a yolk sac in the uterus detected by transvaginal ultrasonography, including ectopic pregnancy, after confirmation of a positive HCG test. Early miscarriage was defined as spontaneous abortion of an intrauterine pregnancy before 12 weeks of gestation. Late miscarriage was defined as spontaneous abortion of an intrauterine pregnancy from 12 weeks of gestation to 28 weeks of gestation. Ectopic pregnancy was defined as at least one sac outside the uterine cavity. And, pregnancy loss included biochemical pregnancy, miscarriage, and ectopic pregnancy. Ongoing pregnancy was defined as the pregnancy exists more than 12 weeks of gestation. A live birth was defined as the delivery of at least one live-birth baby after 28 weeks of pregnancy.

### Statistical analysis

For continuous variables, the data were presented as mean±SD or median (quartile), and then analyzed by the ANOVA test or the non-parametric test. Categorical data were expressed as percentages and compared using the chi-squared. The total measured variables in Table 1, and Tables 2 and 3 were analyzed using R language (version 4.3.2), and furtherly analyzed by survey package with an adjustment of IPW. The inverse probability weights were defined as the reciprocal of propensity scores, and standardized mean difference (SMD) of <10% was considered to be well-balanced covariates among three groups. The univariate logistic regressions and stratified logistic regressions were analyzed by SPSS 27.0 (SPSS IBM Corp., Armonk, NY, USA). Univariate logistic regressions were performed to evaluate the confounding factors. Based on statistical significance, the results of univariate logistic regressions, stratified logistic regressions were further conducted to determine the group allocations affecting the outcomes of IVF/ICSI. A value of *P *< 0.05 was considered statistically significant.

**Table 1.** Basic characteristics of D3 FETs and conditions of D3 transferred embryos among the three groups before and after inverse probability weighting.

	D3 FET entire population					D3 FET IPW				
	Compaction group	Non-change group	Expansion group	*P*	SMD	Compaction group	Non-change group	Expansion group	*P*	SMD
**No. of D3 FET cycles, n (%)**	608 (6.5)	4021 (42.8)	4761 (50.7)			610.8 (6.5)	4025.5 (42.9)	4756.2 (50.6)		
**Age (years), mean ± SD**	34.04 ± 5.39	33.68 ± 5.22	33.39 ± 5.00	0.002**	0.083	33.59 ± 5.31	33.57 ± 5.21	33.58 ± 4.97	0.994	0.003
**Duration of infertility (years), mean±SD**	3.20 ± 2.62	3.61 ± 2.80	3.82 ± 3.00	<0.001***	0.148	3.79 ± 3.35	3.69 ± 2.88	3.68 ± 2.88	0.870	0.023
**BMI (kg/m^2^), mean ± SD**	22.31 ± 3.22	22.23 ± 3.23	22.05 ± 3.25	0.014*	0.054	22.21 ± 3.16	22.15 ± 3.21	22.16 ± 3.29	0.906	0.013
**No. of previous ET times**										
0	254 (41.8)	1889 (47.0)	2325 (48.8)	<0.001***	0.128	268.1 (43.9)	1819.5 (45.2)	2244.9 (47.2)	0.149	0.086
1	177 (29.1)	1229 (30.6)	1423 (29.9)			182.6 (29.9)	1256.1 (31.2)	1398.3 (29.4)		
2	177 (29.1)	903 (22.5)	1013 (21.3)			160.1 (26.2)	949.9 (23.6)	1113.0 (23.4)		
**On the day of progesterone administration**										
Endometrial thickness (mm)	10.02 ± 1.93	9.82 ± 1.82	9.32 ± 1.43	<0.001***	0.275	9.54 ± 1.72	9.57 ± 1.71	9.57 ± 1.61	0.997	0.011
E2 (pmol/l)	1867 (1042–5114)	1553 (941–3636)	1371 (837–2683)	<0.001***	0.317	1841 (1051–5007)	1687 (1021–3785)	1498 (896–2783)	<0.001***	0.205
Progesterone (nmol/l)	1.41 ± 0.99	1.36 ± 0.93	1.41 ± 0.97	0.520	0.035	1.42 ± 0.98	1.40 ± 0.93	1.42 ± 0.91	0.785	0.017
**Conditions of D3 embryos transferred:**										
**No. of D3 embryos transferred, n (%)**										
1	72 (11.8)	276 (6.9)	369 (7.8)	<0.001***	0.123	45.4 (7.4)	306.8 (7.6)	359.5 (7.6)	0.978	0.018
2	528 (86.8)	3705 (92.1)	4357 (91.5)			561.3 (91.9)	3682.1 (91.5)	4353.9 (91.5)		
3	8 (1.3)	40 (1.0)	35 (0.7)			4.2 (0.7)	36.6 (0.9)	42.8 (0.9)		
**Quality of D3 embryos transferred, n (%)**										
1 Grade I embryo at least	555 (91.3)	3655 (90.9)	4321 (90.8)	0.504	0.059	557.0 (91.2)	3651.2 (90.7)	4304.4 (90.5)	0.337	0.041
1 Grade II embryo at least	48 (7.9)	295 (7.3)	354 (7.4)			49.5 (8.1)	305.9 (7.6)	366.2 (7.7)		
All Grade III embryos	5 (0.8)	71 (1.8)	86 (1.8)			4.3 (0.7)	68.4 (1.7)	85.6 (1.8)		
**No. of good-quality D3 embryos transferred, n (%)**										
0	53 (8.7)	366 (9.1)	440 (9.2)	0.913	0.049	53.8 (8.8)	374.3 (9.3)	451.8 (9.5)	0.304	0.037
1	170 (28.0)	1059 (26.3)	1261 (26.5)			174.7 (28.6)	1006.4 (25.0)	1260.4 (26.5)		
2	385 (63.3)	2590 (64.4)	3051 (64.1)			381.1 (62.4)	2640.8 (65.6)	3034.5 (63.8)		
3	0 (0.0)	6 (0.1)	9 (0.2)			1.2 (0.2)	4.0 (0.1)	9.5 (0.2)		

FET, frozen-thawed embryo transfer; ET, embryo transfer; E2, estradiol; Good-quality D3 embryo included grade I D3 embryo; IPW, inverse probability weighting; SMD, standardized mean difference;

* : *P* < 0.05,

** : *P* < 0.01, and

*** : *P* < 0.001.

**Table 2.** Basic characteristics of D5/D6 FETs and conditions of transferred embryos among the three groups before and after inverse probability weighting.

	D5/D6 FET Entire population					D5/D6 FET IPW				
	Compaction group	Non-change group	Expansion group	*P*	SMD	Compaction group	Non-change group	Expansion group	*P*	SMD
**No. of FET cycles, n (%)**	532 (7.5)	3033 (42.9)	3498 (49.5)			535.2 (7.6)	3033.1 (42.9)	3496.3 (49.5)		
**Age (years), mean ± SD**	32.56 ± 4.45	32.30 ± 4.60	32.09 ± 4.50	0.036*	0.070	32.33 ± 4.42	32.24 ± 4.58	32.26 ± 4.52	0.916	0.013
**Duration of infertility (years), mean ± SD**	3.51 ± 2.77	3.35 ± 2.50	3.47 ± 2.55	0.097	0.041	3.42 ± 2.69	3.42 ± 2.56	3.42 ± 2.50	0.998	0.001
**BMI (kg/m^2^), mean ± SD**	22.24 ± 3.19	22.10 ± 3.23	22.00 ± 3.26	0.195	0.050	22.12 ± 3.16	22.07 ± 3.21	22.07 ± 3.29	0.933	0.012
**No. of previous ET times**										
0	241 (45.3)	1493 (49.2)	1819 (52.0)	0.019*	0.093	247.8 (46.3)	1458.9 (48.1)	1741.2 (49.8)	0.350	0.056
1	166 (31.2)	896 (29.5)	1000 (28.6)			161.6 (30.2)	931.2 (30.7)	1017.4 (29.1)		
2	125 (23.5)	644 (21.2)	679 (19.4)			125.8 (23.5)	643.0 (21.2)	737.7 (21.1)		
**On the day of progesterone administration**										
Endometrial thickness (mm)	10.01 ± 2.05	9.81 ± 1.76	9.31 ± 1.41	<0.001***	0.272	9.52 ± 1.74	9.57 ± 1.66	9.57 ± 1.60	0.789	0.020
E2 (pmol/l)	1484 (694–3998)	1288 (660–2922)	1027 (546–2255)	<0.001***		1581 (704–4108)	1368 (721–3021)	1173 (621–2652)	<0.001***	0.197
Progesterone (nmol/l)	1.45 ± 1.0	1.46 ± 1.06	1.53 ± 0.98	0.019*	0.063	1.46 ± 0.98	1.45 ± 0.99	1.51 ± 0.98	0.042*	0.037
**Condition of D5/D6 embryos transferred:**										
**No. of embryos transferred, n (%)**										
1	417 (78.4)	2445 (80.6)	2813 (80.4)	0.485	0.037	432.6 (80.8)	2437.8 (80.4)	2814.4 (80.5)	0.968	0.008
2	115 (21.6)	588 (19.4)	685 (19.6)			102.6 (19.2)	595.3 (19.6)	681.9 (19.5)		
**Stage of embryos transferred, n (%)**										
Day 5	468 (88.0)	2683 (88.5)	3081 (88.1)	0.875	0.010	469.9 (87.8)	2690.4 (88.7)	3076.7 (88.0)	0.650	0.017
Day 6	64 (12.0)	350 (11.5)	417 (11.9)			65.3 (12.2)	342.7 (11.3)	419.6 (12.0)		
**Quality of blastocysts transferred, n (%)**										
1 good-quality blastocyst at least	147 (27.6)	832 (27.4)	997 (28.5)	0.154	0.073	150.4 (28.1)	812.8 (26.8)	1006.9 (28.8)	0.234	0.061
1 moderate-quality blastocyst at least	182 (34.2)	949 (31.3)	1030 (29.4)			171.8 (32.1)	919.0 (30.3)	1010.1 (28.9)		
All low-grade blastocyst	203 (38.2)	1252 (41.3)	1471 (42.1)			213.0 (39.8)	1301.3 (42.9)	1479.3 (42.3)		
**No. of good-quality blastocysts transferred, n (%)**										
0	385 (72.4)	2201 (72.6)	2501 (71.5)	0.379	0.044	384.8 (71.9)	2220.3 (73.2)	2489.4 (71.2)	0.216	0.058
1	143 (26.9)	822 (27.1)	975 (27.9)			145.6 (27.2)	800.7 (26.4)	989.5 (28.3)		
2	4 (0.8)	10 (0.3)	22 (0.6)			4.8 (0.9)	12.1 (0.4)	17.5 (0.5)		

FET, frozen-thawed embryo transfer; ET, embryo transfer; E2, estradiol; Three quality categories for blastocyst: good- (AB, AB, or BA), moderate- (BB), and low-grade (grade C for inner cell mass or trophectoderm); IPW, inverse probability weighting; SMD, standardized mean difference;

* : *P* < 0.05,

** : *P* < 0.01, and

*** : *P* < 0.001.

**Table 3.** Pregnancy outcomes and logistic regressions of D3 FETs and D5/D6 FETs among the three groups after inverse probability weighting.

Pregnancy Outcomes	Compaction group (C)	Non-change group (N)	Expansion group (E)	P1	OR1 (C/N)	P2	OR2 (E/N) unadjusted	P3
**No. of D3 FET cycles, n (%)**	610.8 (6.5)	4025.5 (42.9)	4756.2 (50.6)					
**HCG-positive rate per ET, % (n)**	55.8 (340.6)	58.0 (2335.3)	60.7 (2886)	0.012*	0.912 (0.759–1.096)	0.327	1.117 (1.024–1.218)	0.013*
**Clinical pregnancy rate per ET, % (n)**	46.8 (286.1)	48.8 (1962.9)	52.5 (2496.8)	<0.001***	0.926 (0.780–1.097)	0.369	1.161 (1.068–1.264)	<0.001***
**Ongoing pregnancy rate per ET, % (n)**	38.0 (232.4)	40.6 (1634.4)	44.2 (2104.3)	<0.001***	0.898 (0.754–1.070)	0.229	1.162 (1.066–1.265)	0.001**
**Live birth rate per ET, % (n)**	36.7 (224)	39.6 (1594.1)	43.3 (2060.2)	<0.001***	0.883 (0.731–1.067)	0.199	1.166 (1.070–1.271)	0.001**
**Pregnancy loss rate per HCG-positive case, % (n)**	34.2 (116.6)	31.7 (741.2)	28.6 (825.8)	0.018*	1.120 (0.869–1.441)	0.383	0.862 (0.764–0.973)	0.016*
Biochemical pregnancy rate	12.5 (42.5)	13.4 (312.2)	11.9 (342.7)	0.273	0.924 (0.657–1.302)	0.654	0.873 (0.739–1.031)	0.110
Early miscarriage rate	15.8 (53.7)	14.1 (328.5)	13.6 (392.5)	0.567	1.143 (0.816–1.597)	0.437	0.962 (0.818–1.130)	0.633
Late miscarriage rate	2.5 (8.4)	1.7 (40.3)	1.5 (44.1)	0.448	1.439 (0.657–3.145)	0.363	0.885 (0.573–1.366)	0.581
Ectopic pregnancy rate	3.5 (12)	2.6 (60.2)	1.6 (46.5)	0.015*	1.381 (0.704–2.710)	0.348	0.619 (0.418–0.917)	0.017*
**Live birth rate per HCG-positive case, % (n)**	65.8 (224)	68.3 (1594.1)	71.4 (2060.2)	0.018*	0.894 (0.693–1.151)	0.383	1.160 (1.028–1.309)	0.016*
**No. of D5/D6 FET cycles, n (%)**	535.2 (7.6)	3033.1 (42.9)	3496.3 (49.5)					
**HCG-positive rate per ET, % (n)**	61.8 (330.6)	66.5 (2018.1)	68.9 (2407.2)	0.001**	0.813 (0.668–0.989)	0.039*	1.112 (1.010–1.236)	0.049*
**Clinical pregnancy rate per ET, % (n)**	53.4 (285.7)	55.4 (1681.0)	58.0 (2029.3)	0.031*	0.920 (0.765–1.106)	0.375	1.113 (1.008–1.227)	0.033*
**Ongoing pregnancy rate per ET, % (n)**	44.4 (237.7)	46.3 (1402.8)	48.0 (1677.6)	0.176	0.928 (0.771–1.116)	0.427	1.072 (0.972–1.181)	0.163
**Live birth rate per ET, % (n)**	42.8 (229.3)	45.4 (1377.8)	47.1 (1647.5)	0.130	0.901 (0.743–1.091)	0.285	1.071 (0.97–1.182)	0.176
**Pregnancy loss rate per HCG-positive cases, % (n)**	30.6 (101.3)	31.7 (640.3)	31.6 (759.7)	0.930	0.951 (0.733–1.233)	0.702	0.992 (0.87–1.129)	0.907
Biochemical pregnancy rate	12.9 (42.6)	15.9 (320.7)	15.2 (366.9)	0.370	0.782 (0.552–1.110)	0.169	0.951 (0.80–1.122)	0.555
Early miscarriage rate	14.5 (48.0)	13.8 (278.2)	14.6 (351.7)	0.740	1.062 (0.753–1.499)	0.732	1.071 (0.90–1.272)	0.441
Late miscarriage rate	2.50 (8.3)	1.20 (24.9)	1.20 (30.0)	0.140	2.096 (0.948–4.630)	0.068	1.012 (0.59–1.739)	0.964
Ectopic pregnancy rate	0.7 (2.3)	0.80 (16.4)	0.5 (11.0)	0.370	0.847 (0.187–3.846)	0.830	0.561 (0.25–1.244)	0.155
**Live birth rate per HCG-positive case, % (n)**	69.4 (229.3)	68.3 (1377.8)	68.4 (1647.5)	0.930	1.052 (0.812–1.364)	0.702	1.008 (0.88–1.147)	0.907

ET, embryo transfer; FET, frozen-thawed embryo transfer; OR, odds ratio; OR1, odds ratio for pregnancy outcomes comparing the compaction group to the non-change group (reference); OR2, odds ratio for pregnancy outcomes comparing the expansion group to the non-change group (reference); P1, the statistical difference on pregnancy results among three groups; P2, the statistical difference on the odds ratios of pregnancy outcomes between compaction group and non-change group; P3, the statistical difference on the odds ratios of pregnancy outcomes between expansion group and non-change group;

* : *P* < 0.05,

** : *P* < 0.01, and

*** : *P* < 0.001.

## Results

**Figure 1.**
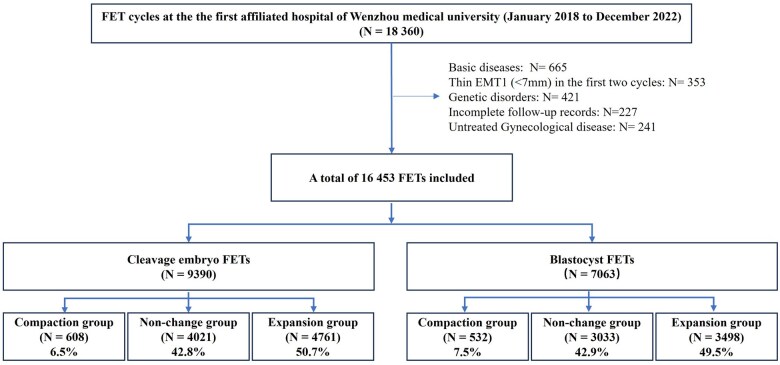
**Study flow chart.** If EMT1 was less than 7 mm but thicker than in any previous fresh or FET cycles, these cycles were still included in the present study. Untreated gynecological diseases included hydrosalpinx, uterine polyps, submucosal fibroids, severe adenomyosis, uterine fibroids (>5 cm), uterine malformations. FET, frozen-thawed embryo transfer; EMT1, endometrial thickness at the end of proliferative phase. Basic diseases included hypertension, diabetes, immune diseases.

Figure 1 shows that 9390 D3 FETs and 7063 D5/D6 FETs cycles were included, and divided into three groups as follows: (i) in D3 FETs, 608 (6.5%) cycles in the compaction group, 4021 (42.8%) cycles in the non-change group, and 4761 (50.7) cycles in the expansion group (Fig. 1 and Table 1) and (ii) in D5/D6 FETs, 532 (7.5%) cycles in the compaction group, 3033 (42.9%) cycles in the non-change group, and 3498 (49.5%) cycles in the expansion group (Fig. 1 and Table 2).

### Baseline characteristics

The baseline characteristics of D3 and D5/D6 FETs before and after IPW are shown in Tables 1 and 2. After IPW, no significant differences were observed in female age, duration of infertility, BMI, the number of previous failed FETs, or embryo number and quality. Estradiol levels were the highest in the compaction group and the lowest in the expansion group in both D3 FETs and D5/D6 FETs (*P *< 0.001). In D5 FETs, progesterone levels significantly differed among the three groups, but the differences showed no clinical meanings (*P *= 0.042, SMD = 0.037).

### Pregnancy outcomes in D3 and D5/D6 FET cycles

Pregnancy outcomes of D3 and D5/D6 FETs before and after IPW are presented in Supplementary Table S1 and Table 3. Logistic regressions with IPW adjustment were further conducted to evaluate the predictive value of group allocations on pregnancy outcomes.

In D3 FETs, after IPW adjustment, significant differences were observed among the three groups (compaction group vs non-change group vs expansion group) in HCG-positive rates per ETs (55.8% vs 58.0% vs 60.7%; *P *= 0.012), clinical pregnancy rates per ETs (46.8% vs 48.8% vs 52.5%; *P *< 0.001), ongoing pregnancy rates per ETs (38.0% vs 40.6% vs 44.2%; *P *< 0.001), and LBRs per ETs (36.7% vs 39.6% vs 43.3%; *P *< 0.001). Among HCG-positive cases, the compaction group had the highest ectopic pregnancy rates (3.5% vs 2.6% vs 1.6%; *P *= 0.015) and the lowest LBRs (65.8% vs 68.3% vs 71.4%; *P *= 0.018), while no significant differences were found in biochemical pregnancy rates, early miscarriage rates, and late miscarriage rates (*P *> 0.05). Logistic regressions with IPW found that the expansion group was weakly associated with higher odds of HCG-positive chances (OR 1.117, 95% CI: 1.024–1.218; *P *= 0.013), clinical pregnancy chances (OR1.161, 95% CI: 1.068–1.264; *P *< 0.001), ongoing pregnancy chances (OR 1.162, 95% CI: 1.066–1.265; *P *= 0.001), live birth chances per ETs (OR 1.166, 95% CI: 1.070–1.271; *P *= 0.001), and live birth chances per HCG-positive cases (OR 1.160, 95% CI: 1.028–1.309; *P *= 0.016) compared to the non-change group. The expansion group also had lower odds of ectopic pregnancy chances per HCG-positive cases (OR 0.619, 95% CI: 0.418–0.917, *P *= 0.017) compared to the non-change group. However, the compaction group showed no significant association with any pregnancy outcomes compared to the non-change group (*P *> 0.05).

In D5/D6 FETs, after IPW adjustment, significant differences were observed among the three groups (compaction group vs non-change group vs expansion group) in HCG-positive rates per ETs (61.8% vs 66.5% vs 68.9%; *P *= 0.001), and clinical pregnancy rates per ETs (53.4% vs 55.4% vs 58.0%; *P *= 0.031). However, no significant differences were found in ongoing pregnancy rates per ETs, LBRs per ETs, LBRs per HCG-positive cases, and pregnancy loss rates per HCG-positive cases (*P *> 0.05). Logistic regressions with IPW adjustment found that the expansion group was weakly associated with higher odds of HCG-positive chances (OR 1.112, 95% CI: 1.010–1.236; *P *= 0.049), and clinical pregnancy chances (OR 1.113, 95% CI: 1.008–1.227; *P *= 0.033) compared to the non-change group. The compaction group had lower odds of HCG-positive chances (OR 0.813, 95% CI: 0.668–0.989, *P *= 0.039), but no significant difference in clinical pregnancy odds (*P *> 0.05) compared to the non-change group. Neither the expansion nor compaction group showed significant association with ongoing pregnancy chances per ETs, live birth chances per ETs, and live birth chances per HCG-positive cases, pregnancy loss chances per HCG-positive cases (*P *> 0.05) compared to the non-change group.

### Factors associated with LBRs per ETs

To investigate factors associated with LBRs in D3 and D5/D6 FETs, univariate logistic regression analyses were performed (Table 4). In D3 FETs, female age (OR 0.903, 95% CI: 0.895–0.911), the number of transferred embryos (OR 1.89, 95% CI: 1.617–2.209), EMT1 (OR 1.036, 95% CI: 1.011–1.062), EMT2 (OR 1.054, 95% CI: 1.032–1.077), and EMT change ratios (OR 1.007, 95% CI: 1.004–1.011) were weakly associated with LBRs. In D3 FETs, females with compressed endometrium had lower live birth chances (OR 0.831, 95% CI: 0.696–0.993), while those with expanded endometrium had higher live birth chances (OR 1.168, 95% CI: 1.073–1.272), compared to those with non-changed endometrium. In D5 FETs, female age (OR 0.929, 95% CI: 0.919–0.939), the number of transferred embryos (OR 1.129, 95% CI: 1.003–1.269), EMT2 (OR 1.035, 95% CI: 1.010–1.061), and EMT change ratios (OR 1.004, 95% CI: 1.000–1.008) were weakly associated with LBRs.

**Table 4.** Factors associated with live birth rates in D3 and D5/D6 FETs.

	Univariate (D3 FET)			Univariate (D5 FET)		
	OR unadjusted	95% CI		OR unadjusted	95% CI	
**Age**	**0.903**	0.895	0.911	**0.929**	0.919	0.939
**No. of embryos transferred**	**1.890**	1.617	2.209	**1.129**	1.003	1.269
**EMT1**	**1.036**	1.011	1.062	1.027	0.998	1.057
**EMT2**	**1.054**	1.032	1.077	**1.035**	1.010	1.061
**EMT change ratios**	**1.007**	1.004	1.011	**1.004**	1.000	1.008
**EMT groups**						
Compaction group	**0.831**	0.696	0.993	0.897	0.745	1.080
Non-change group	1.000			1.000		
Expansion group	**1.168**	1.073	1.272	1.075	0.976	1.186

EMT, endometrial thickness; EMT1, endometrial thickness at the end of proliferative phase; EMT2, endometrial thickness on embryo transfer day; FET, frozen-thawed embryo transfer; OR, odds ratio; Odds ratios in bold indicate statistically significant differences (*P* < 0.05).

### Stratified analysis of factors associated with LBRs in D3 and D5/D6 FETs

**Figure 2.**
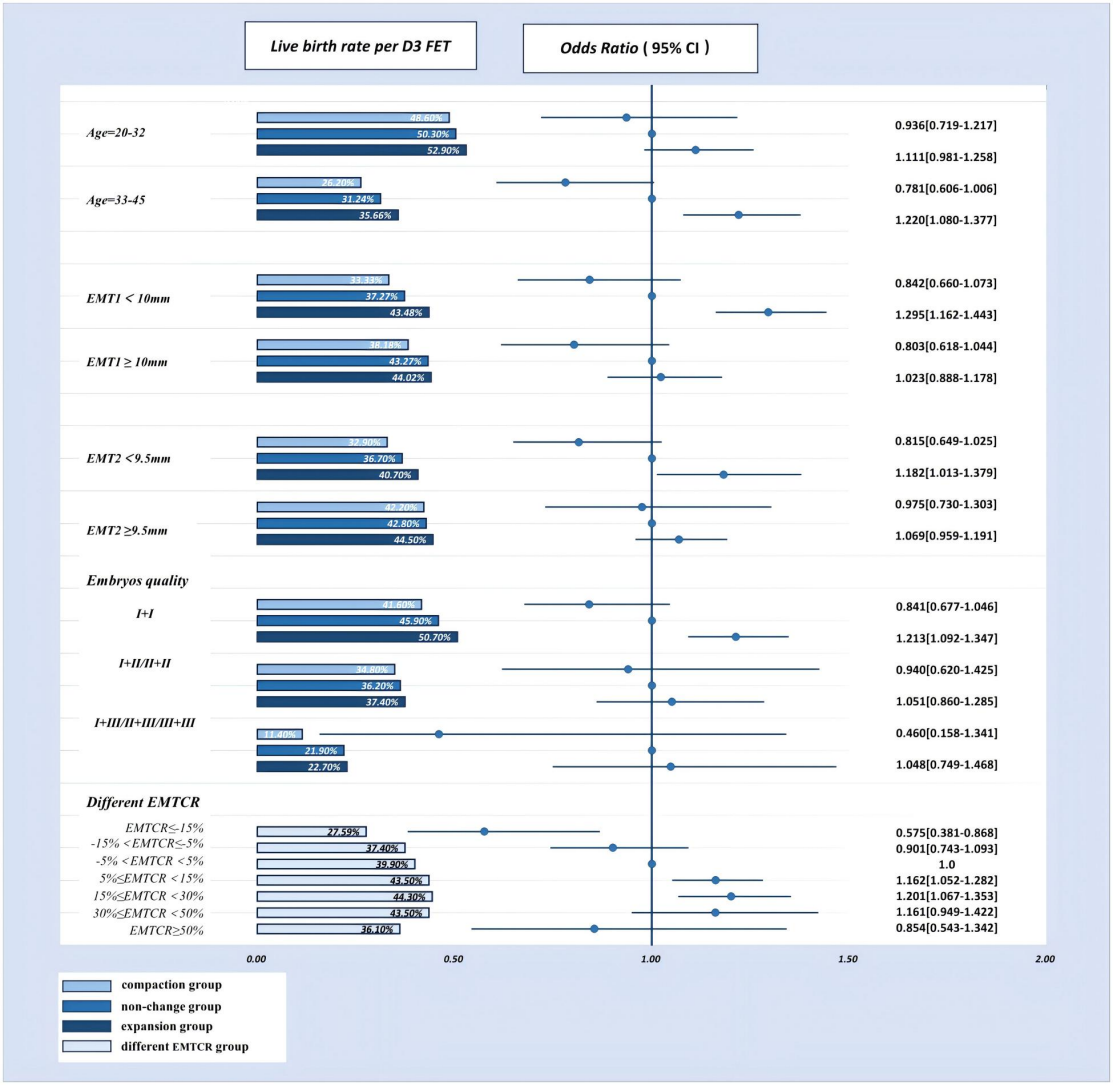
**Stratified logistic regressions of live birth rates in D3 FETs among three groups after inverse probability weighting.** The non-change group is regarded as the reference. The quality of embryos at cleavage stage was graded according to the Istanbul consensus (Alpha Scientists in Reproductive Medicine and ESHRE Special Interest Group of Embryology, 2011). Embryo transfers were performed with two embryos, and the analyses were stratified by the following combinations: I+I, I+II or II+II, and I+III or II+III or III+III. FET, frozen-thawed embryo transfer; EMT1, endometrial thickness at the end of proliferative phase; EMT2, endometrial thickness on the day of embryo transfer; EMTCR, endometrial thickness change ratio.

**Figure 3.**
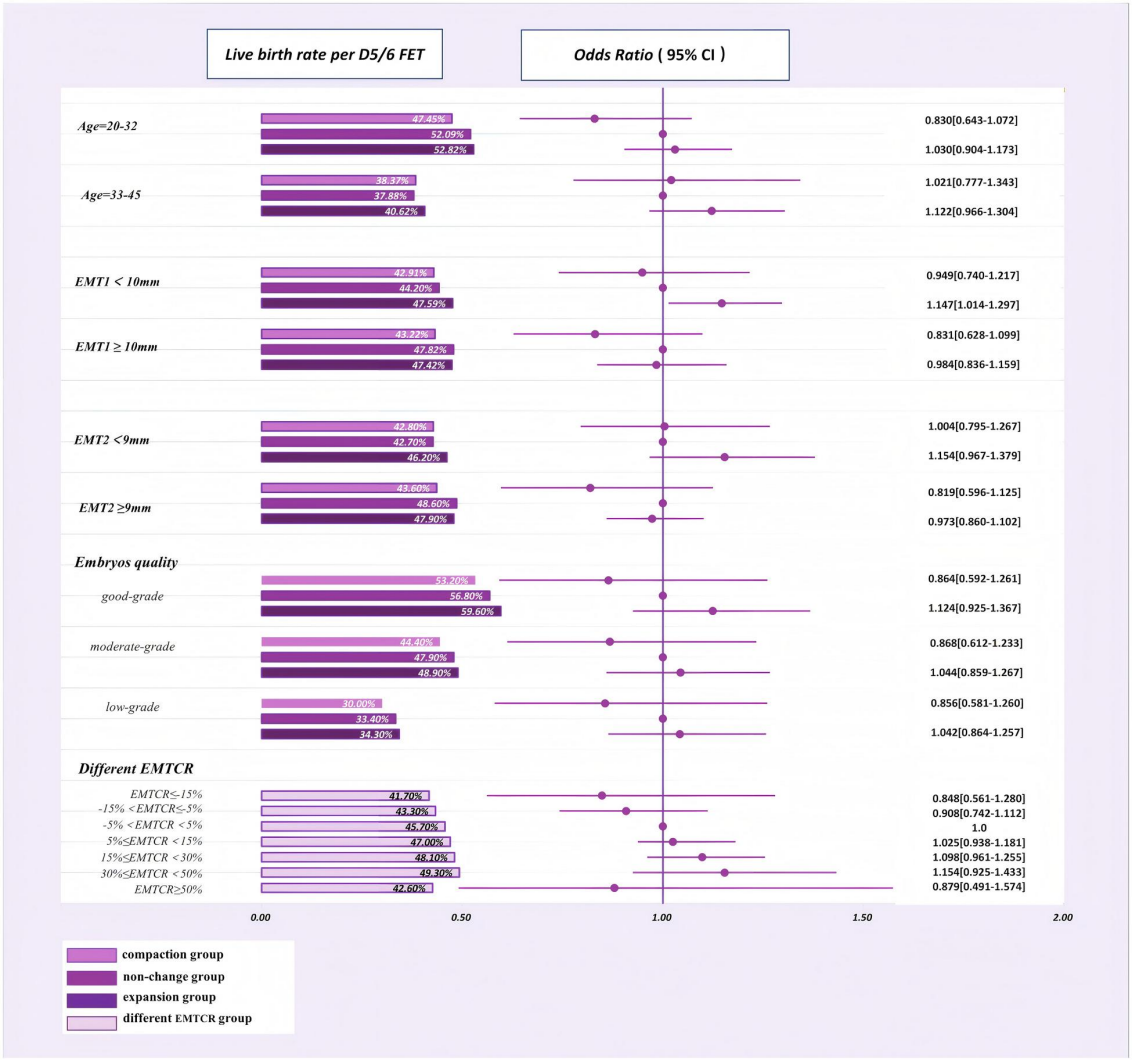
**Stratified logistic regressions of live birth rates in D5/D6 FETs among three groups after inverse probability weighting.** The non-change group is regarded as the reference. The quality of blastocysts was assessed according to Gardner scoring (Alpha Scientists in Reproductive Medicine and ESHRE Special Interest Group of Embryology, 2011). Blastocysts were classified with an A or B in both inner cell mass (ICM) and trophectoderm (TE) as good-grade blastocysts (AA, AB, or BA), those with a B in both ICM and TE as moderate-grade blastocysts (BB), and those with a C in either ICM or TE as low-grade blastocysts (AC, CA, BC, CB, CC) (Zou *et al.*, 2023). FET, frozen-thawed embryo transfer; EMT1, endometrial thickness at the end of proliferative phase; EMT2, endometrial thickness on the day of embryo transfer; EMTCR, endometrial thickness change ratio.

Based on the factors associated with LBRs per ETs, D3 FETs (Fig. 2) and D5/D6 FETs (Fig. 3) were further stratified by female age, EMT1, EMT2, the conditions of embryos transferred (the number and the quality), and EMT change ratios, and analyzed.

Figure 2 shows the results of the stratified LBR analyses and logistic regression analyses for D3 FETs. Women more than 32 years old with expanded endometrium had significantly higher LBRs per ETs (35.7% vs 31.2%) than those in the non-change group (OR 1.220, 95% CI: 1.080–1.377, *P *= 0.001). When EMT1 was less than 10 mm, the expansion group showed significantly higher LBRs per ETs (43.5% vs 37.3%) than the non-change group (OR 1.295, 95% CI: 1.162–1.443, *P *< 0.001). When EMT2 was less than 9.5 mm, the expansion group had significantly higher LBRs per ETs (40.7% vs 36.7%) than the non-change group (OR 1.182, 95% CI: 1.013–1.379, *P *= 0.034). The expansion group with two Grade I embryos transferred had significantly higher LBRs per ETs (50.7% vs 45.9%) than the non-change group (OR 1.213, 95% CI: 1.092–1.347, *P *< 0.001). LBRs were significantly increased with the increase of EMT change ratios, when EMT change ratio was less than 30%. Women with EMT change ratios less than −15% had significantly lower LBRs per ETs (27.6% vs 39.9%) than those with non-changed endometrium (OR 0.575, 95% CI: 0.381–0.868, *P *= 0.008). Women with EMT change ratios of 5–15% (OR 1.162, 95% CI: 1.052–1.282, *P *= 0.030), or 15–30% (OR 1.201, 95% CI: 1.067–1.353, *P *= 0.020) were more likely to have live births than those with non-changed endometrium. When compared to women with non-changed endometrium, women with EMT change ratios higher than 50% or ranged from −5% to −15% had lower LBRs per ETs, though the differences were not statistically significant.

Figure 3 presents the stratified results of LBR analyses and logistic regression analyses for D5/D6 FETs. When EMT1 was less than 10 mm, the expansion group had significantly higher LBRs per ETs (47.6% vs 44.2%) than the non-change group (OR 1.147, 95% CI: 1.014–1.297, *P *= 0.030). LBRs of D5/D6 FET cycles stratified by female age, EMT2, and the quality of transferred embryos were not affected significantly by EMT group allocations. When EMT change ratios were less than 50%, LBRs per ETs of D5/D6 FETs were positively associated with EMT change ratios, though the differences were not statistically significant.

## Discussion

This retrospective study of 16 453 hormone replacement FETs revealed that compacted endometrium significantly decreased HCG-positive rates per ETs and clinical pregnancy rates per ETs compared to non-changed or expanded endometrium in both D3 and D5/D6 FETs. Univariate logistic regressions found that LBRs were weakly associated with compacted endometrium. However, endometrial compaction occurred in a very small fraction of cycles. Stratified logistic regressions showed that the beneficial effects of expanded endometrium on LBRs may be associated with the following factors, including embryo developmental stages, maternal age, EMT change ratios, and EMT at the day of transformation/ET.

Our study revealed endometrial compaction rates of 6.5% in D3 and 7.5% in D5/D6 FET cycles (Tables 1 and 2), lower than the 14.6–45% range reported in some previous studies (Haas *et al.*, 2019; Zilberberg *et al.*, 2020; Riestenberg *et al.*, 2021; Shah *et al.*, 2022; Ju *et al.*, 2023). These differences likely arise from four factors. First, endometrial compaction definitions differed substantially across studies, with threshold ranging from 0% to 10% EMT reduction (Haas *et al.*, 2019; Jin *et al.*, 2021a,b; Kaye *et al.*, 2021; Olgan *et al.*, 2022). Second, while previous studies predominantly employed transabdominal ultrasound for assessment prior to transfer (Haas *et al.*, 2019; Zilberberg *et al.*, 2020; Shah *et al.*, 2022; Ju *et al.*, 2023), we utilized more accurate transvaginal ultrasound throughout cycles. Third, critical timing variations existed in the measurements of EMT1 (Haas *et al.*, 2019; Huang *et al.*, 2020; Ye *et al.*, 2020; Zilberberg *et al.*, 2020; Jin *et al.*, 2021a,b; Olgan *et al.*, 2022; Shah *et al.*, 2022) and EMT2 (ET day vs preceding day) (Riestenberg *et al.*, 2021). Finally, endometrial preparation protocols varied markedly between studies, including natural cycles (Huang *et al.*, 2020; Jin *et al.*, 2021a; Youngster *et al.*, 2022), fresh cycles (Huang *et al.*, 2021; Li *et al.*, 2022), or hormonal protocols (Haas *et al.*, 2019; Zilberberg *et al.*, 2020; Riestenberg *et al.*, 2021; Olgan *et al.*, 2022; Ju *et al.*, 2023). Notably, although our measurements were conducted by experienced clinicians using triplicate assessments, inter-operator variability during same-cycle evaluations may contribute to observed differences. These methodological divergences in definition criteria, imaging modality, temporal measurements, and preparation protocols collectively lead to the differences in endometrial compaction rates across studies.

EMT dynamics differ fundamentally between natural and hormonal replacement cycles. In natural cycles, post-ovulatory endometrial thickening occurs under coordinated estrogen–progesterone regulation (Das *et al.*, 1997; Dockery *et al.*, 1998), though secondary estrogen surges post-corpus luteum formation create distinct hormonal patterns. By contrast, hormone-replacement cycles maintain stable estrogen levels during progesterone administration. Our findings demonstrate significantly higher estradiol concentrations in women with endometrial compaction compared to non-change or expansion groups (Tables 1 and 2), suggesting that supraphysiological estrogen levels during the transformation phase may disrupt hormonal balance and endometrial remodeling. Indeed, Bu *et al.* (2019) showed that compressed endometrium occurred more frequently in natural cycles than in hormonal replacement ones. While moderate estradiol concentrations adequately support endometrial maturation (Sugiura *et al.*, 2018), excessive estrogen exposure before progesterone administration does not sustain dose-dependent endometrial growth (Deng *et al.*, 2018). Elevated estradiol levels not only fail to promote dose-dependent endometrial growth but may compromise receptivity through mechanisms including glandular apoptosis, as evidenced by *in vitro* models (Valbuena *et al.*, 1999; Joo *et al.*, 2010; Chen *et al.*, 2014). This paradoxical effect likely explains the ultrasonographic appearance of compressed endometrium in high-estrogen scenarios. The observed endometrial thickening post-progesterone administration in FET cycles aligns with these pathophysiological mechanisms, representing appropriate physiological responsiveness to hormonal regulation. These observations highlight the delicate estrogen–progesterone interplay in endometrial preparation. The increased compaction frequency in natural cycles may reflect inherent hormonal fluctuations absent in controlled replacement protocols. These findings emphasize the need for optimized estrogen dosing strategies to balance endometrial growth with receptivity requirements in ART cycles.

The present study found that in D3 FETs, ≥15% endometrial compaction significantly reduced LBRs, while D5 FETs showed a non-significant trend. Expansion ≥50% trended toward lower LBRs in all stages of embryo (Figs 2 and 3). This pattern likely reflects two key determinants. First, embryo viability influences implantation capacity differentially across developmental stages (Wu *et al.*, 2020). Second, the optimal EMT range (7–14 mm) correlates with favorable pregnancy outcomes (El-Toukhy *et al.*, 2008; Aydin *et al.*, 2013; Ugwu *et al.*, 2022). In this study, mean EMT2 values of 6.6 mm (with compression ≥15%) and 14 mm (with expansion ≥50%), both exceeded this optimal range. Thus, clinicians should assess the pregnancy outcomes through EMT change ratios combining with EMT and embryo quality.

Embryo quality may contribute to the contradictory findings regarding endometrial compaction’s predictive value for pregnancy outcomes (Haas *et al.*, 2019; Zilberberg *et al.*, 2020; Youngster *et al.*, 2022; Ju *et al.*, 2023; Yaprak *et al.*, 2023). While studies using euploid embryos, or non-stratified embryos reported varied associations, our analysis of non-biopsied embryos categorized by developmental stage revealed distinct patterns. Notably, our studies further found that D3 ETs exhibited significant positive correlations between LBRs and EMT change ratios, unlike D5/D6 transfers (Figs 2 and 3), suggesting embryo developmental potential rather than EMT changes may determine LBRs. Elevated estradiol levels in the compaction group may impair cleavage-stage embryo development in *in vitro* culture, reducing blastocyst formation and quality (Valbuena *et al.*, 2001). These findings imply that expanded endometrium in FET cycles may favor superior pregnancy outcomes for D3 ET, while developmental competence dominated outcomes in D5/D6 ET.

There were several advantages and limitations in the present study. This retrospective study was the largest sample research, employing stratified analyses adjusting for maternal age, embryo quality, embryonic development stage, EMT on the day of transformation/transfer. All FET cycles followed standardized endometrial preparation with transvaginal ultrasound monitoring. The main limitations of this study were as follows: (i) This study only included untested embryos for FETs, as the certification for preimplantation genetic diagnosis was not obtained during the period of the study; (ii) Although triplicate EMT measurements minimized inter-observer variability, a 5% change in EMT might lie within the potential variability due to the inter- and intra-observer variability; (iii) This study was retrospective, and thus prospective studies should be investigated to record precisely and to avoid memory bias; (iv) The first two cycles with EMT1 less than 7 mm were not included in the analyses. These patients constitute a high-risk subgroup necessitating prioritized clinical surveillance; (v) Some basic characteristics were missing, such as infertility etiology and uterine/endometrial configuration. To eliminate confounding factors, we excluded patients with abnormal uteri or endometrium, as these conditions are considered to have poor pregnancy outcomes.

## Conclusion

In summary, our study demonstrated that endometrial compaction after progesterone administration is not associated with better pregnancy outcomes. LBRs increased with the increase of EMT within a specific range, with distinct optimal ranges for D3 and D5/D6 FETs. Notably, the assessment of endometrial changes may be less predictive of LBRs in D5/D6 FETs. Moreover, the negative impact of endometrial compaction on pregnancy outcomes is more pronounced in D3 FETs. Therefore, different endometrial changes are required for FETs at different developmental stages, necessitating different clinical decisions.

## Supplementary Material

hoaf039_Supplementary_Data

## Data Availability

All data generated or analyzed during this study are included in this published article. The datasets used and/or analyzed during the current study are available from the corresponding author on reasonable request.
