# Supplementary material for: Endometrial compaction shows no association with improved pregnancy outcomes in hormonal replacement frozen-thawed embryo transfer: an analysis of over 16 000 cases
Source: Hum Reprod Open. 2025 Jun 20;2025(3):hoaf039. doi: 10.1093/hropen/hoaf039 (PMC12255888; doi:10.1093/hropen/hoaf039)
Supplement: hoaf039_Supplementary_Data [file hoaf039_supplementary_data.docx]

**Supplementary Table S1 Pregnancy outcomes and logistic regressions of D3 FETs and D5/D6 FETs among three groups before inverse probability weighting.**

| **Pregnancy Outcomes** | **Compaction group (C)** | **Non-change group (N)** | **Expansion group (E)** | **P1** | **OR1 (C/N)** | **P2** | **OR2 (E/N) unadjusted** | **P3** |
| --- | --- | --- | --- | --- | --- | --- | --- | --- |
| **No. of D3 FET cycles, n (%)** | 608 (6.5) | 4021 (42.8) | 4761 (50.7) |  |  |  |  |  |
| **HCG positive rate per ET, % (n)** | 54.8 (333) | 58.2 (2342) | 60.8 (2897) | 0.003****** | 0.868 (0.731-1.031) | 0.106 | 1.114 (1.023-1.214) | 0.013***** |
| **Clinical pregnancy rate per ET, % (n)** | 45.2 (275) | 49.1 (1971) | 52.7 (2508) | <0.001******* | 0.859 (0.724-1.019) | 0.082 | 1.158 (1.064-1.259) | <0.001******* |
| **Ongoing pregnancy rate per ET, % (n)** | 36.8 (224) | 40.9 (1644) | 44.6 (2122) | <0.001******* | 0.843 (0.707-1.006) | 0.058 | 1.163 (1.068-1.266) | <0.001******* |
| **Live birth rate per ET, % (n)** | 35.5 (216) | 39.9 (1603) | 43.6 (2078) | <0.001******* | 0.831 (0.696-0.993) | 0.041**^*^** | 1.168 (1.073-1.272) | <0.001******* |
| **Pregnancy loss rate per HCG positive case, % (n)** | 35.1 (117) | 31.6 (739) | 28.3 (819) | 0.004****** | 1.175 (0.923-1.496) | 0.190 | 0.855 (0.759-0.963) | 0.010****** |
| Biochemical pregnancy rate | 14.1 (47) | 13.4 (314) | 11.8 (341) | 0.143 | 1.061 (0.763-1.477) | 0.724 | 0.862 (0.731-1.015) | 0.075 |
| Early miscarriage rate | 15.3 (51) | 14.0 (327) | 13.3 (386) | 0.546 | 1.114 (0.809-1.535) | 0.507 | 0.947 (0.808-1.110) | 0.503 |
| Late miscarriage rate | 2.4 (8) | 1.8 (41) | 1.5 (44) | 0.440 | 1.381 (0.642-2.973) | 0.409 | 0.866 (0.564-1.329) | 0.509 |
| Ectopic pregnancy rate | 3.3 (11) | 2.4 (57) | 1.7 (48) | 0.038***** | 1.369 (0.711-2.639) | 0.347 | 0.675 (0.458-0.995) | 0.047***** |
| **Live birth rate per HCG positive case, % (n)** | 64.9 (216) | 68.4 (1603) | 71.7 (2078) | 0.004****** | 0.851 (0.669-1.083) | 0.190 | 1.170 (1.039-1.317) | 0.010 |
| **No. of D5/D6 FET cycles, n (%)** | 532(7.5) | 3033 (42.9) | 3498(49.5) |  |  |  |  |  |
| **HCG positive rate per ET, % (n)** | 62.2 (331) | 67.0 (2032) | 69.2 (2419) | 0.003****** | 0.811 (0.670-0.982) | 0.032**^*^** | 1.104 (0.995-1.226) | 0.062 |
| **Clinical pregnancy rate per ET, % (n)** | 53.6 (285) | 55.8 (1693) | 58.3 (2039) | 0.037***** | 0.913 (0.759-1.099) | 0.336 | 1.106 (1.001-1.220) | 0.044**^*^** |
| **Ongoing pregnancy rate per ET, % (n)** | 44.7 (238) | 46.6 (1412) | 48.4 (1693) | 0.152 | 0.929 (0.772-1.119) | 0.438 | 1.077 (0.970-1.187) | 0.137 |
| **Live birth rate per ET, % (n)** | 43.0 (229) | 45.7 (1387) | 47.5 (1663) | 0.091 | 0.897 (0.745-1.080) | 0.251 | 1.075 (0.976-1.186) | 0.143 |
| **Pregnancy loss rate per HCG positive cases, % (n)** | 30.8 (102) | 31.7 (645) | 31.3 (756) | 0.912 | 0.958 (0.745-1.231) | 0.737 | 0.978 (0.861-1.110) | 0.978 |
| Biochemical pregnancy rate | 13.3 (44) | 15.9 (324) | 15.3 (369) | 0.443 | 0.808 (0.576-1.134) | 0.218 | 0.949 (0.807-1.116) | 0.527 |
| Early miscarriage rate | 14.2 (47) | 13.8 (281) | 14.3 (346) | 0.901 | 1.031 (0.739-1.439) | 0.856 | 1.040 (0.878-1.232) | 0.650 |
| Late miscarriage rate | 2.7 (9) | 1.2 (25) | 1.2 (30) | 0.100 | 2.244 (1.038-4.851) | 0.040**^*^** | 1.008 (0.591-1.720) | 0.976 |
| Ectopic pregnancy rate | 0.6 (2) | 0.7 (15) | 0.5 (11) | 0.471 | 0.817 (0.186-3.591) | 0.789 | 0.614 (0.282-1.340) | 0.221 |
| **Live birth rate per HCG positive case, % (n)** | 69.2 (229) | 68.3 (1387) | 68.7 (1663) | 0.912 | 1.044 (0.812-1.342) | 0.737 | 1.023 (0.901-1.161) | 0.726 |

ET, embryo transfer; FET, frozen-thawed embryo transfer; OR, odds ratio; OR1, odds ratio for pregnancy outcomes comparing the compaction group to the non-change group (reference); OR2, odds ratio for pregnancy outcomes comparing the expansion group to the non-change group (reference); P1, the statistical difference on pregnancy results among three groups; P2, the statistical difference on the odd ratios of pregnancy outcomes between compaction group and non-change group; P3, the statistical difference on the odd ratios of pregnancy outcomes between expansion group and non-change group; *: P < 0.05, **: P < 0.01 and ***: P < 0.001.
